# Trio-Drug Combination of Sodium Valproate, Baclofen and Thymoquinone Exhibits Synergistic Anticonvulsant Effects in Rats and Neuro-Protective Effects in HEK-293 Cells

**DOI:** 10.3390/cimb44100299

**Published:** 2022-09-20

**Authors:** Faheem Hyder Pottoo, Mohammed Salahuddin, Firdos Alam Khan, Batool Taleb Albaqshi, Mohamed S. Gomaa, Fatima S. Abdulla, Noora AlHajri, Mohammad N. Alomary

**Affiliations:** 1 Department of Pharmacology, College of Clinical Pharmacy, Imam Abdulrahman Bin Faisal University, P.O. Box 1982, Dammam 31441, Saudi Arabia; 2 Department of Clinical Pharmacy Research, Institute for Research and Medical Consultation, Imam Abdulrahman Bin Faisal University, P.O. Box 1982, Dammam 31441, Saudi Arabia; 3 Department of Stem Cell Research, Institute for Research and Medical Consultation, Imam Abdulrahman Bin Faisal University, P.O. Box 1982, Dammam 31441, Saudi Arabia; 4 Department of Pharmaceutical Chemistry, College of Clinical Pharmacy, Imam Abdulrahman Bin Faisal University, P.O. Box 1982, Dammam 31441, Saudi Arabia; 5 College of Medicine and Health Science, Khalifa University, Abu Dhabi P.O. Box 127788, United Arab Emirates; 6 Department of Medicine, Sheikh Shakhbout Medical City (SSMC), Abu Dhabi P.O. Box 127788, United Arab Emirates; 7 National Centre for Biotechnology, Kind Abdulaziz City for Science and Technology (KACST), Riyadh 11442, Saudi Arabia

**Keywords:** epilepsy, sodium valproate, baclofen, thymoquinone, HEK-293 cells, neuroprotection, PI3K/Akt/mTOR pathway

## Abstract

Epilepsy is a chronic brain disorder, with anti-epileptic drugs (AEDs) providing relief from hyper-excitability of neurons, but largely failing to restrain neurodegeneration. We investigated a progressive preclinical trial in rats, whereby the test drugs; sodium valproate (SVP; 150 and 300 mg/kg), baclofen (BFN; 5 and 10 mg/kg), and thymoquinone (THQ; 40 and 80 mg/kg) were administered (i.p, once/day for 15 days) alone, and as low dose combinations, and subsequently tested for antiseizure and neuroprotective potential using electrical stimulation of neurons by Maximal electroshock (MES). The seizure stages were monitored, and hippocampal levels of m-TOR, IL-1β, IL-6 were measured. Hippocampal histopathology was also performed. Invitro and Insilco studies were run to counter-confirm the results from rodent studies. We report the synergistic effect of trio-drug combination; SVP (150 mg/kg), BFN (5 mg/kg) and THQ (40 mg/kg) against generalized seizures. The Insilco results revealed that trio-drug combination binds the Akt active site as a supramolecular complex, which could have served as a delivery system that affects the penetration and the binding to the new target. The potential energy of the ternary complex in the Akt active site after dynamics simulation was found to be −370.426 Kcal/mol, while the supramolecular ternary complex alone was −38.732 Kcal/mol, with a potential energy difference of −331.694 Kcal/mol, which favors the supramolecular ternary complex at Akt active site binding. In addition, the said combination increased cell viability by 267% and reduced morphological changes induced by Pentylenetetrazol (PTZ) in HEK-293 cells, which indicates the neuroprotective property of said combination. To conclude, we are the first to report the anti-convulsant and neuroprotective potential of the trio-drug combination.

## 1. Introduction

Seizures result from an imbalance in electric brain impulses, and are characterized by psychiatric manifestations [1,2,3]. Meta-analysis studies conducted from 1985 to 2013 found that incidences of seizures are higher in both adulthood and the beginning of adolescence. Seizures are more frequent in males than females, and are more frequent in low-income societies than in high income societies. The incidence rate of epilepsy varies from 33 to 57 per 100,000 per year [4,5]. The prevalence of seizures is 1%, with long-term remission in more than 50% of the patients with seizures, when completing antiepileptic courses of therapy [5]. The electroshock model is a useful tool to assess the preclinical therapeutic effect of AEDs. It is used to mimic generalized type seizures [6,7,8]. 

Sodium valproate (SVP) is a broad-spectrum antiepileptic drug (AED) that causes alterations in the levels of dopamine (DA), norepinephrine (NE), serotonin (5-HT), and Gamma-aminobutyric acid (GABA) [9]. However, combination therapy is sometimes recommended to improve the effectiveness and tolerance of SVP. Combinations of SVP and docosahexaenoic acid result in increased potency (3.6-fold) of SVP in pentylenetetrazol (PTZ) and kindling seizure models [10]. SVP and Lutein in combination showed a significant decline in seizure score in pilocarpine-induced epilepsy in rats [11]. SVP and TP427 in combination exhibited synergistic effects against epilepsy [12]. SVP and citicoline (nootropic agent) exhibited synergism effects against acute generalized convulsions induced in rats via PTZ [13]. Since, SVP (300 mg/kg/day) is known to distort hepatic lobular architecture with nuclei aggregations [14], we sought to combine SVP at lower doses with Baclofen (BFN) and Thymoquinone (THQ). 

Baclofen (BFN) is a centrally acting skeletal muscle relaxant, a GABA-B-receptor agonist [15]. BFN (10^−8^ M) reduced epileptiform discharges of CA3 pyramidal neurons within hippocampal slices from guinea pigs [16]. BFN (2 microM) reduced the frequency of short ‘interictal’ bursts [17]. Progabide (GABA-A and GABA-B receptor agonist) reduced convulsion severity more than BFN (GABA-B receptor agonist) in a kindling epilepsy model in rats [18]. BFN exhibited anticonvulsant effects in PTZ-induced seizures in rats [19]. Invitro studies have proven that BFN also exhibits anti-inflammatory characteristics via the interruption of proinflammatory chemokine receptors and their function [20]. BFN further reduces IL-6 and IL-12 release in LPS-induced inflammation in mouse microglial cells [21]. BFN reduced IL-6 and TNF-α release and NF-κB and p38 MAPK production in astrocytes and microglia during the inflammatory process stimulated by lipopolysaccharide (LPS) and interferon-γ [22]. 

Thymoquinone (THQ) is a neuroprotective drug [23,24]. THQ is protective against MPP (+) and rotenone-induced damage in primary dopaminergic neurons. THQ also decreased neurotoxicity and apoptosis induced by Aβ1-40 [25]. THQ reduced brain injury caused by status epilepticus (SE) by increasing the expression of Nrf2, SOD, and HO-1 proteins in the hippocampus [26]. THQ reduced the severity of seizures in a lithium–pilocarpine rat model of SE via reducing NF-κB gene expression [27]. The combination of vitamin C and THQ showed positive outcomes against seizures via decreasing high-grade seizures and prolonging seizure onset in PTZ-models by the activation of the GABAB1R/CaMKII/CREB pathway [28]. The anticonvulsant effect of THQ is also reported against penicillin-induced epileptiform activity in rats [29]. THQ has an anti-inflammatory and neuroprotective effect in CPF-induced neuronal injury in rats. The possible anti-inflammatory effect manifests as the reduction of the inflammatory cytokines IL-6 and IL-1β [30]. Another study supports the evidence of a THQ mediated anti-inflammatory effect via TNF-α and cytokine level reduction [31].

The mTOR (mammalian target of rapamycin) signaling pathway is critical for most cell functions such as cell growth, protein synthesis, synaptic elasticity, and proliferation [32,33]. mTOR mutations are highly linked with dysplasia, epilepsy, and neurodevelopment disorders [34]. Hyperactivation of the mTOR pathway contributes to drug-resistant epilepsy that renders it a target for intervention [35,36]. Vigabatrin treatment decreases mTOR pathway activation in *Tsc1*^GFAP^CKO and control astrocytes [37].

Current management of epilepsy involves monotherapy, add-on therapy, monotherapy substitution, and polytherapy [38]. Approximately 60% of epilepsy patients are treated with monotherapy successfully, but when it fails, polytherapy is usually considered. Polytherapy can also be used when titrating a new adjunctive AED towards monotherapy [39]. However, very few ideal combinations have been identified [39]. Before the introduction of many new AEDs in the 1990s, only first-generation Na+ channel blockers existed, and the combination of these led to an amplified risk of side effects. New drugs such as gabapentin, lamotrigine, levetiracetam, and topiramate had variable modes of action, leading to a reduced risk of amplified effects [39]. The rise of these drugs led to the philosophy of rational polytherapy, which suggests combining AEDs of different mechanisms for increased effectiveness and reduced risks in the management of refractive epilepsy [40,41,42]. Thus we thought to combine SVP with BFN and THQ to restrict seizures and provide adequate neuroprotection, thereby intercepting the process of epileptogenesis by interfering with the mTOR signaling pathway and inflammatory cascades.

## 2. Materials and Methods

### 2.1. Ethics Statement

Care and utilization of 120 adult wistar rats (230–260 g, aged 12–14 weeks) was conducted according to protocol from the Institutional Animal Care and Use Committee (IACUC) of IAU after approval (Approval# IRB-2021-05-126). Behavioral experiments were conducted during daytime (8:00 am to 4:00 pm). 

### 2.2. Drugs and Dosing Schedule

Sodium valproate (SVP; 150 and 300 mg/kg), Baclofen (BFN; 5 and 10 mg/kg) and Thymoquinone (THQ; 40 and 80 mg/kg) were used as test drugs. All drug dosages were based on previous studies. SVP (300 mg/kg) protected against PTZ induced seizures in wistar rats [13]. BFN (5 mg/kg, i.p) was anticonvulsant in the pentylenetetrazol (PTZ)-induced seizures [19]. THQ (40 mg/kg) attenuated PTZ-induced seizures and mortality in rats [28]. The effectiveness of test drugs was studied alone and as low dose combinations. All drug/s were dissolved in 2% Tween 20 and injected intraperitoneally (i.p) to rats (once daily). The dosing continued for 15 days, and electroshock was applied on the last day of treatment. 

### 2.3. Establishment and Assessment of MES Rat Model

Procedures to induce the MES in rats had been described in previous studies [12]. Briefly, electroshock was delivered to rats via auricular electrodes, and the rats were immediately released upon stimulation and kept in a single cage to monitor seizure behavior. Seizure stages were graded by experienced pharmacologists. Tonic hind limb extension (THLE) was taken as the end point. Evaluation continued until the rat’s regained posture.

### 2.4. Study Design

In this experiment, 120 rats were divided into 12 experimental groups (I-XII, n = 10/group) as follows; NC (vehicle 10 mL/kg), TC (vehicle 10 mL/kg), SVP1 (150 mg/kg), SVP2 (300 mg/kg), BFN1 (5 mg/kg), BFN2 (10 mg/kg), THQ1 (40 mg/kg), THQ2 (80 mg/kg), SVP1 + BFN1 (150 + 5 mg/kg), SVP1 + THQ1 (150 + 40 mg/kg), THQ1 + BFN1 (40 + 5 mg/kg), SVP1+ BFN1 + THQ1 (150 + 5 + 40 mg/kg). Seizures were induced on the last day of treatment in rat groups II-XII, brain tissues were collected immediately by sacrificing six rats after their recovery from seizures and then processed for estimation of mTOR, IL-1β and IL-6 in hippocampus, while remaining four rats were sacrificed at 24 h and brain processed for hippocampal histopathology.

### 2.5. Estimation of Hippocampal mTOR, IL-1β and 1L-6 Levels

The levels of hippocampal mTOR, IL-1β and 1L-6 levels were measured by ELISA. The manufacturer’s (AVIVA Biosystem’s, San Diego, CA, USA) instructions were followed. 

### 2.6. Invitro Studies

MTT assay was used to evaluate the cell viability in a 96-well plate seeded with HEK-293 cells in growth medium, cultured for 24 h to obtain 70–80% confluence. These cells were treated with PTZ (0.6 µg/mL) for 24 h followed by treatment with SVP (120 µg/mL), BFN (1.50 µg/mL), THQ (12.0 µg/mL) and SVP + BFN + THQ (120 + 1.50 + 12.0 µg/mL) for another 24 h. Then 10 μL MTT solution was poured into each well. After 4 h, the cell medium was removed and 100 μL/well (5 mg/mL) of DMSO was added. The absorbance was read at 570 nm in a microplate reader and morphological changes were visualized under a phase-contrast microscope.

### 2.7. Molecular Modeling

All molecular modeling studies performed used the molecular operating environment (MOE) 2014.0901 molecular modeling software for molecular docking simulation molecular dynamics and ligand binding energy calculation. Pymol was used for output data visualization and figure generation. Ligand preparation, energy minimization, and potential energy calculation were performed using the MOE interface. The crystal structure of human Akt (PDB code; 4gv1), co-crystallized with an inhibitor, was used as a target in the docking studies. The selected target and ligands were prepared using protein preparation and LigX tools, respectively. All hydrogens were added to the ligand PDB file and partial charges, and ionization state were computed. The docking was performed using MOE dock tool in MOE, performed with the default values. The binding pocket of the co-crystallized ligand was used to define the active site for docking. Molecular dynamic simulation was performed using the dynamics tool in MOE using the NPA algorithm at a temperature of 300º K with a sample time of 0.5 ps for a total frame of 500. The system for dynamic simulation was solvated with a water box with a margin of 10 Å and equilibrated with NaCl. The results were assessed via a binding energy calculation, and by checking ligand binding positioning through any interaction with key residues. Comparison with the crystallized ligand position was also carried out. 

### 2.8. Statistical Analysis

**Figure 1:**
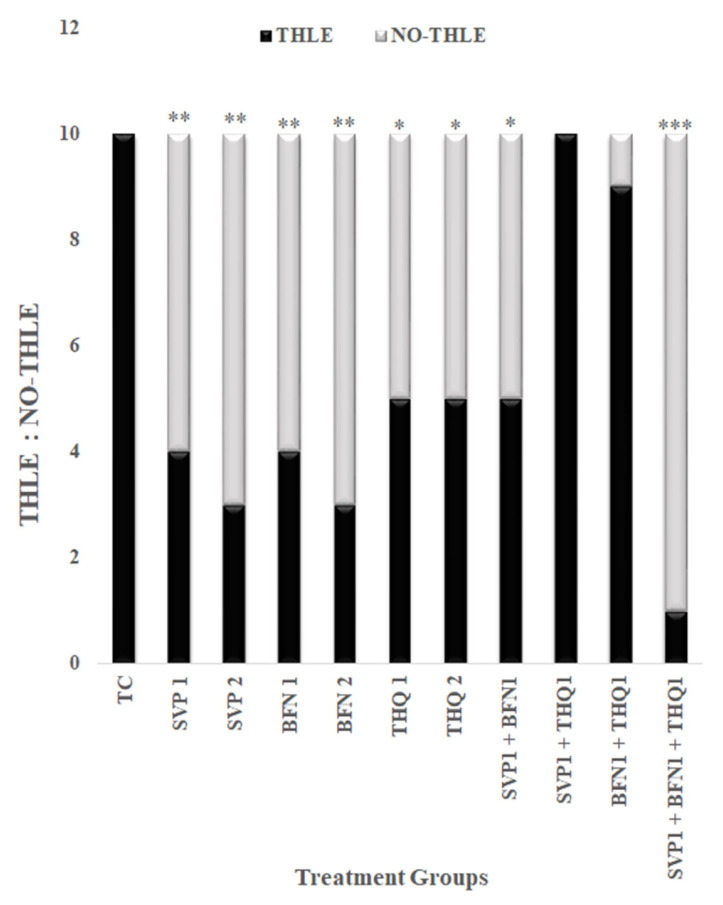
Effect of SVP (150 and 300 mg/kg), BFN (5 and 10 mg/kg), THQ (40 and 80 mg/kg) alone and in low dose combination on MES induced THLE. The values are expressed as ratio of THLE:NO-THLE. * *p* < 0.05, ** *p* < 0.01, *** *p* < 0.001 versus toxic control rats. Fisher’s exact test (2 × 2 contingency table; One tailed) was used for statistical analysis.

**Figure 2:**
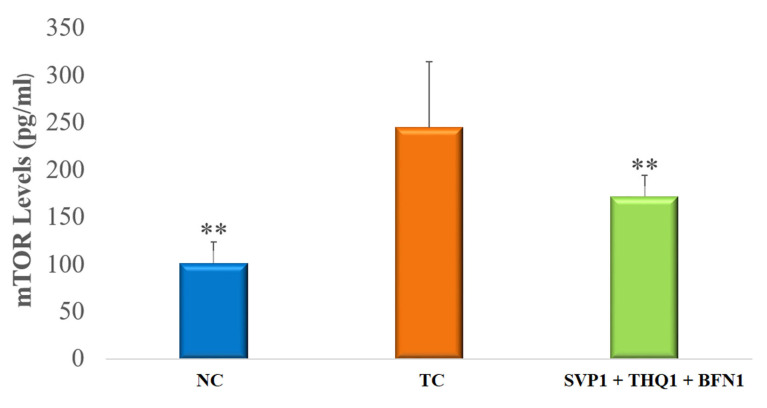
Effect of SVP (150 mg/kg) + BFN (5 mg/kg) + THQ (40 mg/kg) treatment on hippocampal mTOR levels. The values are expressed as mean ± SEM. ** *p* < 0.01 versus TC. NC; Normal Control, TC; Toxic Control.

**Figure 3:**
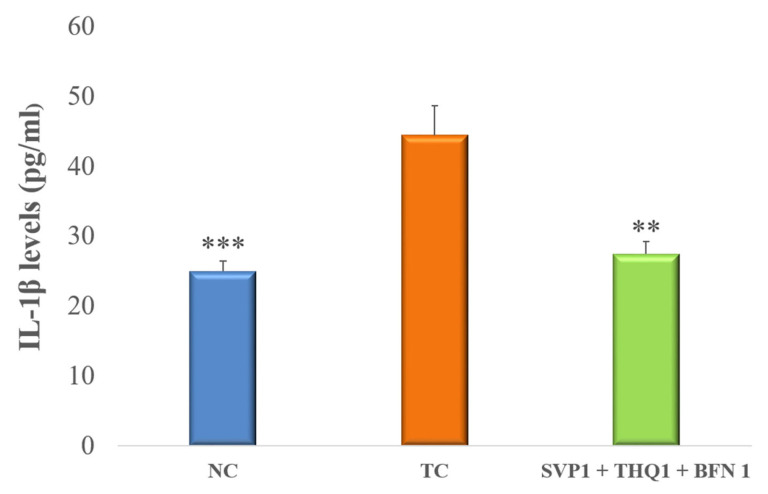
Effect of SVP (150 mg/kg) + BFN (5 mg/kg) + THQ (5 mg/kg) treatment on hippocampal levels of IL-1β. The values are expressed as mean ± SEM. ** *p* < 0.01, *** *p* < 0.001 versus TC. NC; Normal Control, TC; Toxic Control.

**Figure 4:**
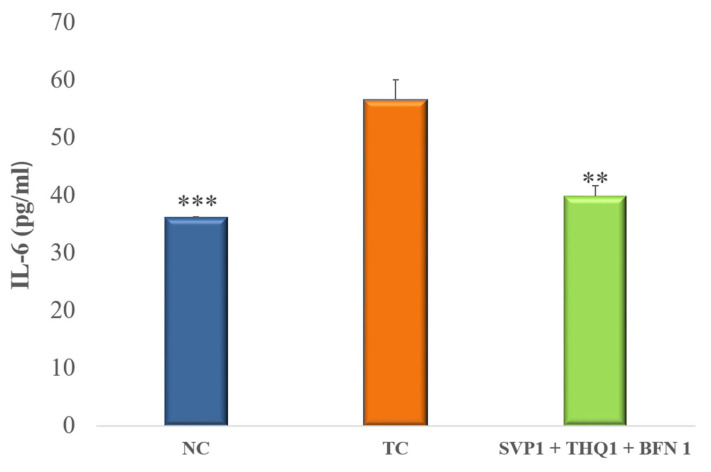
Effect of SVP (150 mg/kg) + BFN (5 mg/kg) + THQ (5 mg/kg) treatment on hippocampal levels of IL-6. The values are expressed as mean ± SEM. ** *p* < 0.01, *** *p* < 0.001 versus TC. NC; Normal Control, TC; Toxic Control.

Data is presented as mean ± SEM, except for data on the ratio of THLE:NO-THLE in rats, which was assessed via Fischer’s exact test (one tailed) (Figure 1). Hippocampal levels of mTOR, IL-1β, and 1L-6 levels were analyzed by ANOVAs followed by post hoc Dunnet’s test (Figures 2–4). The number of surviving hippocampal neurons were calculated by using one-way ANOVAs, followed by Tukey HSD (Figure 6). In case of % cell viability *p*-values were calculated by Student’s *t*-test (Figure 7). All groups were compared with toxic control (TC). *p* < 0.05 was considered statistically significant in all cases. GraphPad InStat was used for analysis. 

## 3. Results

### 3.1. Anticonvulsant Effect of Sodium Valproate (SVP), Baclofen (BFN), Thymoquinone (THQ) Alone and in Combination against MES-Induced Seizures

MES resulted in tonic hind limb extension (THLE) in all rats, monotherapy with SVP (150 and 300 mg/kg) and BFN (5 and 10 mg/kg) very significantly (*p* < 0.01) prevented MES-induced THLE, while THQ (40 and 80 mg/kg) significantly (*p* < 0.05) prevented MES-induced THLE. Combination therapy of SVP (150 mg/kg) and BFN (5 mg/kg) significantly (*p* < 0.05) prevented MES-induced THLE, however the efficacy was less than the individual drugs. The other combinations—THQ (40 mg/kg) and SVP (150 mg/kg), and THQ (40 mg/kg) with BFN (5 mg/kg)—were non-efficacious. Interestingly, the trio-drug combination of SVP (150 mg/kg) with BFN (5 mg/kg) and THQ (40 mg/kg) exhibited an extremely significant (*p* < 0.001) anti-convulsant effect and synergism. These results encourage further investigation of molecular mechanisms involving the anticonvulsant efficacy of low dose combination regimen; SVP (150 mg/kg) + BFN (5 mg/kg) +THQ (40 mg/kg) (Figure 1).

### 3.2. Hippocampal mTOR Levels

In this case, mTOR is critical for the development of seizures. MES-induced seizures in rats exhibited very significant (*p* < 0.01) increases in hippocampal mTOR levels. The trio-drug combination of SVP (150 mg/kg) + BFN (5 mg/kg) + THQ (40 mg/kg) very significantly (*p* < 0.01) reversed the rise in mTOR levels induced by the seizure, compared to Toxic Control (TC) (Figure 2).

### 3.3. Pro-Inflammatory Cytokines

An extremely significant (*p* < 0.001) rise in brain hippocampal levels of IL-1β and IL-6 was observed after the induction of seizures in rats. Treatment with SVP (150 mg/kg) + BFN (5 mg/kg) + THQ (40 mg/kg) very significantly (*p* < 0.01) improved the raised inflammatory response, as evident by a significant decline in the IL-1β and IL-6 levels in the hippocampus, compared to TC. (Figure 3 and Figure 4).

### 3.4. Neuroprotective Effect of Trio-Drug Combination of Sodium Valproate (SVP), Baclofen (BFN), Thymoquinone (THQ) on Rat Hippocampus

**Figure 5:**
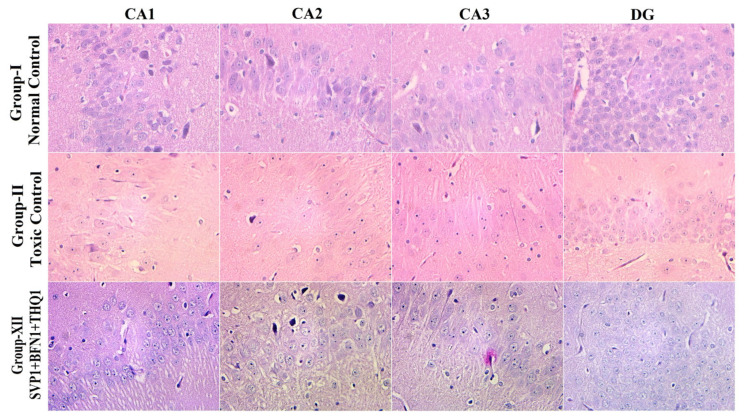
The H/E-stained hippocampal section visualized under 40× shows normal arrangements and distributions in the normal control group (Group-I). Plenty of neuronal loss, degeneration and death from electroshock observed in Group-II. Slight cellular changes in CA1, CA2, CA3, DG of the SVP (150 mg/kg) + BFN (5 mg/kg) + THQ (40 mg/kg) injected rats are indicative of minimal neuronal degeneration and nuclear pyknosis in Group-XII.

**Figure 6:**
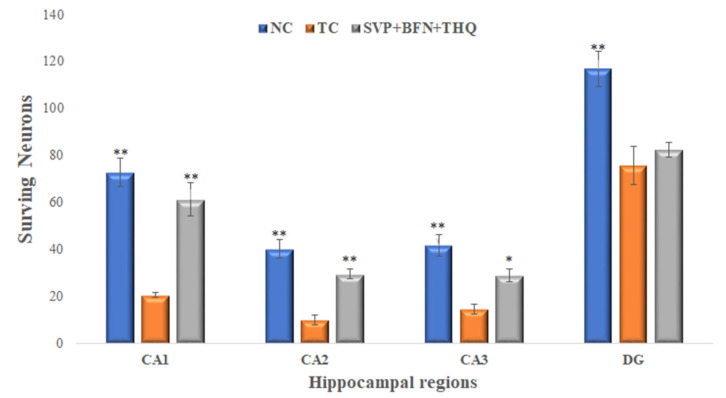
Surviving neurons from hippocampal regions, CA1, CA2, CA3 and DG. * *p* < 0.05, ** *p* < 0.01 versus TC. NC; Normal Control, TC; Toxic Control.

Hematoxylin and eosin staining of rat hippocampi revealed normal arrangements and distributions of neuronal cells in CA1, CA2, CA3 and DG in the normal control group (Group-I). Hippocampal changes in the vehicle-injected rats exposed to electroshock include reduction in neuronal density in all hippocampal regions (Group-II). The SVP (150 mg/kg) + BFN (5 mg/kg) + THQ (40 mg/kg) injected rats revealed very significant protection from neuronal loss in CA1 and CA2 areas, and significant protection in CA3 area (Group-XII) after electroshock magnification ×40 (Figure 5 and Figure 6).

### 3.5. Effect of Sodium Valproate (SVP), Baclofen (BFN), Thymoquinone (THQ) Alone and in Combination on HEK-293 Cell Viability and Cell Morphology—Invitro

**Figure 7:**
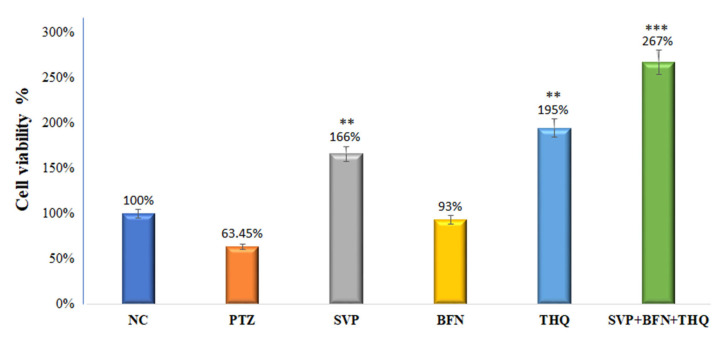
% cell viability of HEK-293 cells by MTT assay. The cells were first exposed to PTZ (0.6 µg/mL), and later with different concentrations of SVP (120 µg/mL), BFN (1.50 µg/mL), THQ (12.0 µg/mL) and SVP + BFN + THQ (120 + 1.5 + 12.0 µg/mL) for 24 hr. The % of cell viability is given in the graph is taken from the dose which gives the highest percentage of cell viability. ** *p* < 0.01, *** *p* < 0.001.

**Figure 8:**
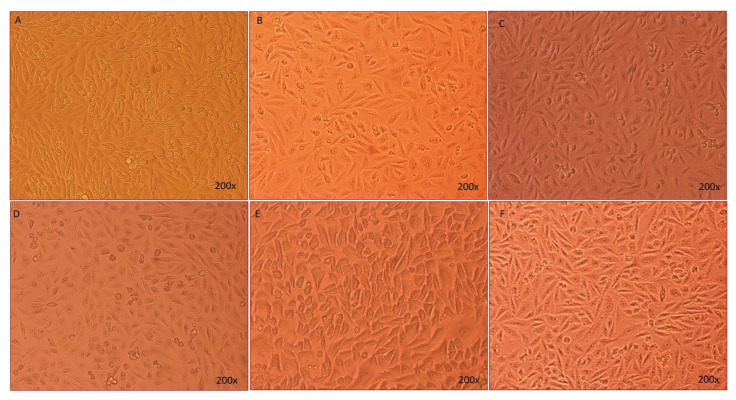
The morphological representation of the HEK-293 cells taken by light microscope. (**A**) Non treated HEK-293 cells; (**B**–**E**) PTZ (0.6 µg/mL), SVP (120 µg/mL), BFN (1.50 µg/mL), THQ (12.0 µg/mL); (**F**) SVP + BFN + THQ (120 + 1.5 + 12.0 µg/mL). The cells were first treated with PTZ for 24 h, then were treated with SVP, BFN and THQ and SVP + BFN + THQ for 24 h. Magnifications 200×.

Figure 7 shows that treatment with SVP (120 µg/mL) and THQ (12 µg/mL) alone increased viability of the HEK-293 cells very significantly (*p* < 0.005) to 166% and 195% resp. BFN (1.50 µg/mL) increased cell viability by 93%, but the effect was not significant. The % cell viability had an extremely significant increase (*p* < 0.0001) to 267% in SVP + BFN + THQ (120 + 12 + 1.5 µg/mL) treated cells. Morphology of the HEK-293 cells was observed after treatments, and Figure 8 shows this compared to control cells, PTZ treatment induced loss of HEK-293 cells, and changes in cell morphology. Together, these results suggest that the trio-drug combination of SVP + BFN + THQ attenuates PTZ-induced toxicity in HEK-293 cells and promotes cellular regeneration (Figure 7 and Figure 8). 

### 3.6. Molecular Simulation Studies

**Figure 9:**
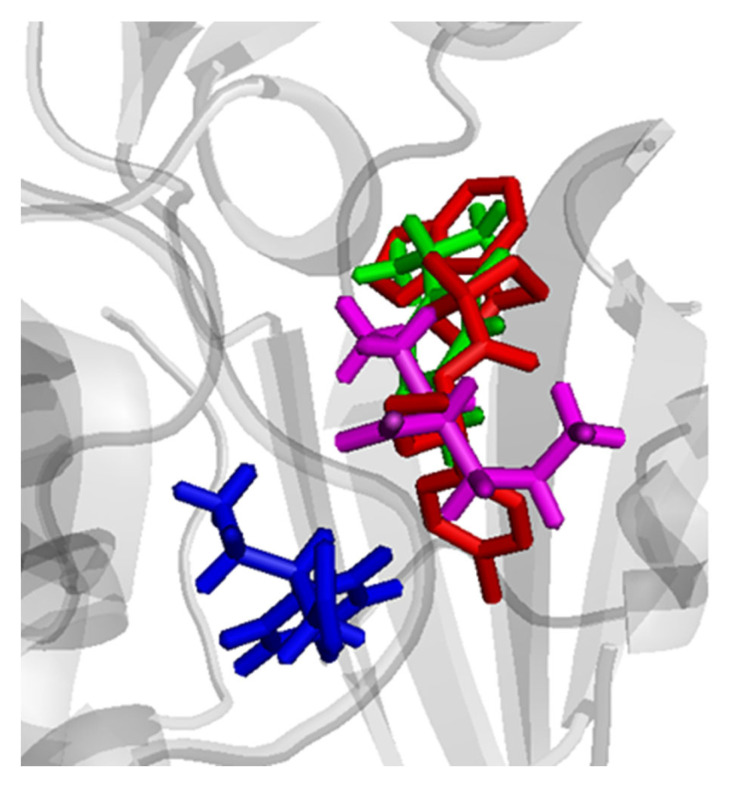
Comparative binding positions of ternary supramolecular complex of the SVP (magenta), BFN (blue) and THQ (green) with the reference crystallized ligand (red) bound to Akt active site.

Molecular simulation was carried out to explain the unpredicted anticonvulsant effect of the trio-drug combination. The fact that the individual drugs showed anticonvulsant activity better than the duo-drug combination/s could be explained based on a supramolecular complex formation. The binary complex could have affected the binding and/or the penetration of the drug to their biological targets. However, in the case of the trio-drug combination, the anti-convulsant activity was not only regained, but was much more superior to the individual drugs. This indicates that the trio-drug combination might have bound a new target in the mTOR pathway that resulted in the synergistic effect. This again could have occurred through a ternary supramolecular complex formation that is able to bind a new specific target in the upstream signaling pathway. Akt, PI3K, and PKRB are potential targets. First, simulation of the supramolecular ternary complex was performed through docking experiments of the three studied drugs, followed by energy minimization and potential energy calculation. The lowest energy supramolecular complex was subjected to molecular dynamic simulation to assess the stability of the complex and search for the lowest possible energy conformation. The potential energy for the lowest energy complex after dynamics was found to be −38.732 Kcal/mol. The three drugs were then sequentially docked in the Akt active site and results were analyzed to find the best docking conformations for the three drugs in terms of binding affinity, overall pose similarity with the crystallized inhibitor and inter-action with key residues. The arrangement of the three drugs in the active site, in comparison with their arrangement in the simulated supramolecular ternary complex, was also considered. In the best bound conformation, the three drugs were positioned in the active site in a way that overlapped the crystallized inhibitor pose (Figure 9). 

**Figure 10:**
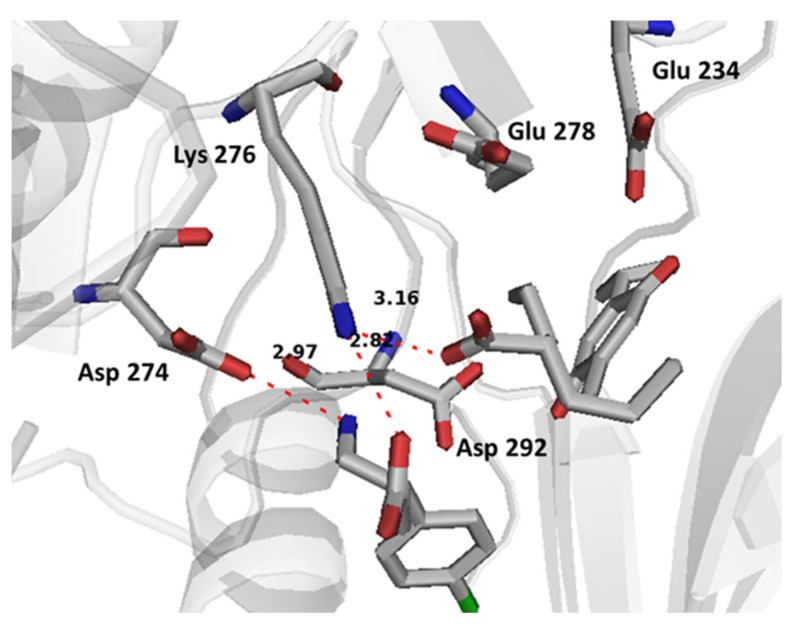
Binding interactions of co-bound ternary supramolecular complex of SVP, BFN and THQ with Akt allosteric site and active site. The distances are represented by red dotted lines, and are measured in Angstrom.

BFN established electrostatic interactions with the key allosteric residue Asp 274 and the active site residue Lys 276 with its ammonium and carboxylate groups, respectively. Bond lengths were 2.97 Å and 2.87 Å, respectively. There was also a potential electrostatic interaction between sodium valproate carboxylate and Lys 276. The docking showed that Lys 276 is a key binding residue that is able to form a salt bridge with BFN and SVP through ionic and charge-assisted hydrogen bonding. The above-mentioned interactions allowed THQ to be positioned strategically between Glu 234 and Asp 292 which it can binds through coordinating water molecules (Figure 10). 

**Figure 11:**
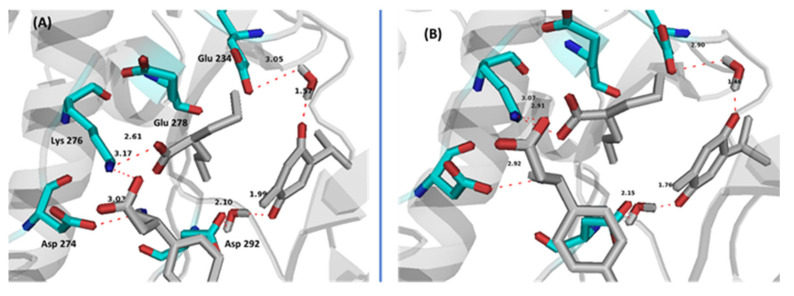
Binding interactions of co-bound ternary supramolecular complex of SVP, BFN and THQ with Akt allosteric site and active site and showing key solvating water molecules. (**A**) first frame in dynamics, (**B**) last frame in dynamics. Distances are represented by red dotted lines and are measured in Angstrom.

Promising results were obtained from molecular dynamic simulation which proved the stability of the supramolecular complex in Akt active/allosteric site. After molecular dynamics, all three drugs kept their positions with non-significant changes in potential intermolecular and supramolecular bond lengths (Figure 11). Two solvating water molecules were found crucial through binding THQ with Glu 234 and Asp 292. These two water molecules kept their strategic positions throughout the dynamic simulation time which again support the stability of the supramolecular complex at the Akt active/allosteric site.

The relatively stable complex can therefore deliver the three drugs to the active site where they cooperatively bind. The supramolecular complex formed could have served as a delivery system that affected the penetration and binding to the new target. The complex is energetically favored to break and bind to the target, and forms a more stable complex in case of the ternary complex. The potential energy of the ternary complex in the Akt active site after dynamics was found to be −370.426 Kcal/mol while in case of the supramolecular ternary complex alone was −38.732 Kcal/mol, with a potential energy difference of −331.694 Kcal/mol that favors the supramolecular ternary complex Akt active site binding.

**Figure 12:**
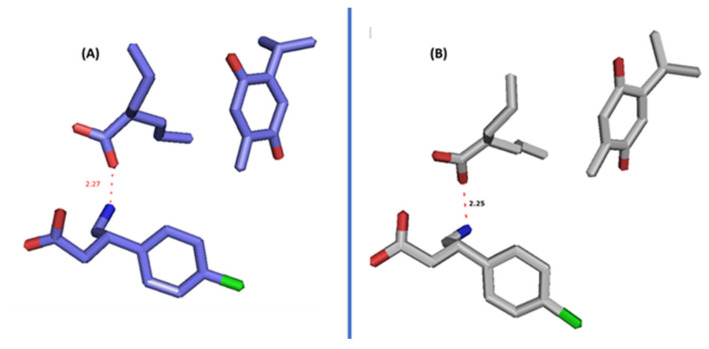
Poses of the three drugs in the supramolecular ternary complex, (**A**) alone and (**B**) after docking in dynamics in Akt enzyme (**B**).

The pose that the three drugs adapted in the active site is also similar to their arrangement in the simulated supramolecular ternary complex (Figure 12). Electrostatic interaction between BFN and SVP and VW interactions between THQ and BFN and SVP stabilize the complex on its own and helps in holding the complex tightly in Akt pockets. This could also mean that the three drugs can also bind the Akt active site in the supramolecular complex form.

## 4. Discussion

Epilepsy is a chronic neurological disorder [43]. It is described by abnormal neurological signs and symptoms due to multiple seizures [44]. Seizures are associated with critical CNS insults such as toxic, metabolic, or structural insults [45]. The combinatorial AED regimens that showed favorable outcomes in human studies are VPA with LTG (lamotrigine), LTG with CBZ (carbamazepine), or PHT (phenytoin). In addition, the combination of VPA with ethosuximide (ETX) showed promising results when used to treat absence seizures in children refractory to ETX as monotherapy. In addition to that, other AEDs that have a positive outcome are LTG-LEV and lacosamide (LCM)-LEV, VPA with lamotrigine (LTG), (LEV) or gabapentin (GBP) [46,47]. Studies have proven the effectiveness of SVP with ETX, or LTG for absence seizures [48]. Nonetheless, one-third of epilepsy cases still remain refractory [46], bringing attention to the possible additional mechanisms contributing to epileptogenesis. Furthermore, the combinations of current AEDs have a higher risk of exaggerating the drug load resulting in brimming drug loading and adverse effects, such as hepatotoxicity and teratogenic effects [49,50,51]. Thus, we combined SVP with allied neuroprotective drugs to enhance the efficacy and reduce the adverse effects. In this present study, we found that the combination of SVP, a broad-spectrum anticonvulsant with BFN (skeletal muscle relaxant) and THQ (neuroprotective) exhibits a synergistic anticonvulsant effect against MES induced seizures. We found that the mechanism of synergism is dependent upon the reduction in levels of mTOR and proinflammatory cytokines in rat hippocampus. Our findings are supported by computational studies which revealed the formation of supramolecular ternary complex formation between SVP, BFN and THQ at the Akt site, stipulating that the PI3K/Akt/mTOR signaling pathway is intercepted at multiple points by the trio-drug combination (this pathway is activated during seizures in animal models, and with pharmacological inhibition of this pathway, epileptiform activity is reduced). Seizures are likely to have an important effect on neuronal survival - we therefore developed an invitro assay to test cell viability. The HEK-293 cells were pretreated with PTZ (which reduced cell viability), while exposure of these pretreated HEK-293 cells with the trio-drug combination resulted in 267% increase in cell viability and also preserved cell morphology. The data therefore implicates, for the first time, a novel synergistic anti-convulsant combination with neuroprotective properties. 

SVP (150 and 300 mg/kg) is an established broad spectrum anticonvulsant drug found to very significantly reduce electrically induced convulsions. BFN (5 and 10 mg/kg) exhibits anticonvulsant effects, which is reported in the literature as well, such as Tyurenkov et al., 2012, 2016 reported reduction in seizure intensity in the MES model [52,53]. Since BFN is a skeletal muscle relaxant, the mechanism for anticonvulsant effects of BFN could involve suppression of synaptic transmission and epileptiform activity [54]. THQ (40 and 80 mg/kg) significantly inhibited convulsions, which is in line with previous studies reporting THQ efficacy against convulsions and other neurological disorders such as anxiety, Parkinsonism, and dementia [25,55]. The animal studies as well have confirmed the anti-ictal effect of THQ. Beyazcicek et al., 2016 reported that THQ reduces the incidence of spike waves and epileptic activity and frequency in penicillin-induced seizures [29], Hosseinzadeh et al., 2005 reported reduction in time to onset of seizures and seizure duration in the PTZ model [56] and Pottoo et al., 2021 reported reduction in THLE following MES induced seizures in THQ treated rats [57]. The duo-drug combinatorial therapy of SVP (150 mg/kg) with BFN (5 mg/kg) exhibited anticonvulsant effect, but the efficacy was no better than monotherapy with individual drugs. Much to the contrary, the combination of THQ (40 mg/kg) with either SVP (150 mg/kg) or BFN (5 mg/kg), altogether abolished the efficacy, which is contrary to findings in literature where THQ is reported to potentiate the potency of SVP in the MES [58] and PTZ model [59]. Nevertheless, the trio-drug combination of SVP (150 mg/kg) with BFN (5 mg/kg) and THQ (40 mg/kg) exhibited synergism in abrogation of MES induced THLE. To elucidate the mechanism, the trio drug combination was tested for its effect on mTOR signaling and pro-inflammatory mediators.

Recent findings have highlighted the dysfunction of the mTOR pathway in epilepsy, and regulating this pathway by multi-drug regimen seems to be a feasible option [60]. The protein is a part of two larger signaling complexes: mTORC1 and mTORC2. PI3K/Akt activation regulates mTORC1 [60]. Gene mutations such as TSC2 and TSC1 contribute to losses in mTORC1 inhibition that cause distribution in synaptogenesis and cell overgrowth [34,61]. Rapamycin, an mTOR inhibitor, prevented seizures and prolonged survival in *Tsc1*^GFAP^CKO mice [62]. Hyperphosphorylation of S6 (a downstream target of mTOR) was markedly inhibited by rapamycin treatment in rats with chronic seizures [63]. Rapamycin (3 and 6 mg/kg) exhibited seizure-suppressing effects in the post-SE rat model for TLE [64]. Thus suppression of the mTOR pathway is anti-epileptic and restores the normal glutamate signaling pathway that renders it a potential target in epilepsy treatment [65,66,67]. In line with previous studies, we found mTOR signaling hyperactivation with electroshock induced seizures, while the trio-drug combination restored the mTOR levels and acted in a manner similar to rapamycin. The molecular modelling revealed the formation of supramolecular ternary complex formation between the trio-drugs SVP, BFN and THQ at the Akt site, this suggests mitigation of signaling through PI3K/Akt/mTOR pathway at multiple points. 

Seizure activity in microglia and astrocytes is highly linked with inflammatory mediators; for example, interleukin-1 β (IL-1 β) and tumor necrosis factor-α (TNF-α) [68]. Another possible mechanism is the IL-1 receptor (R)/TLR pathway that activates the inflammatory process causing neuronal damage [69,70,71]. Activation of these pathways can lead to reduced seizure thresholds and recurrence of seizures [72]. Rats exposed to lipopolysaccharide (LPS) revealed that proinflammatory cytokines, such as IL-1β, TNF-α, and HMGB1, cause seizure manifestation and recurrence [73,74]. Other proinflammatory cytokines such as IL-6 can contribute to inflammation-induced seizures [75,76]. Moreover, IL-6 up-regulation may potentiate levels of other cytokines, for instance, TNF-α, IL-Iβ, IFN-gamma, and IL-17 [77,78]. In addition to that, it has been reported in previous studies that inflammation modulates the GABA neurotransmitter system [79]. IL-6 exhibits inhibitory effect on GABAergic currents of the rat cortex area. The possible MOA is to change the conduction of the GABAA receptor channel [80]. The literature indicates that reducing the proinflammatory cytokines, would normalize GABAergic signaling and intercept generation of seizures and subsequent remission. We found that the trio-drug combination of SVP (150 mg/kg) with BFN (5 mg/kg) and THQ (40 mg/kg) tended to normalize the levels of IL-1β and IL-6, suggesting contributory mechanisms of trio-drug combination to cause seizure halt. 

Neuronal degeneration and cognitive impairment are the most common comorbidities associated with seizures [81]. Neurodegeneration in epilepsy occurs through increased brain permeability and neuronal loss, increasing size, and the number of astrocytes [82]. SVP exhibits neuroprotective effect by reducing the expression of ER stress proteins such as CHOP and GRP78, and by reducing apoptosis of the neuron in the PTZ epilepsy model [83]. However, studies have reported neuroprotection of SVP at higher concentrations, which are much higher than those reported to be safe in these patients [84]. Previous studies had reported the neuroprotective effects of BFN and THQ [85,86,87,88]. Chronic administration of BFN alleviated neuronal damage by regulating the Bcl-2/Bax ratio and increasing the activation of Akt, ERK, and GSK-3β which suppress cyto-destructive autophagy [89]. BFN-induced restoration of GABA*_B_* receptors after an ischemic insult [85]. Baclofen also exhibited the neuroprotective effect by inhibiting CA1 pyramidal cells loss in the hippocampal area and CaM kinase hippocampus reduction [88]. THQ play a role in treating damaged brain neural tissue [90]. THQ exerts neuroprotective properties via reducing the reactive oxygen species (ROS) and mitochondrial membrane potential inhibition in β-amyloid induced neurotoxicity in rats [91]. Histopathological examination of hippocampal sections from normal and toxic control reveals deteriorating effect of electroshock in the form of neuronal loss, degeneration and death from electroshock, particularly in regions CA1, CA2 and CA3. The degenerative changes were minimal in the trio-drug combination treated group; SVP (150 mg/kg) + BFN (5 mg/kg) + THQ (40 mg/kg). In-vitro studies also revealed the ability of the said combination to increase cell viability of PTZ-treated HEK-293 cells by 267%. Various studies in the literature have refuted the relationship of cell viability with neuroprotective efficacy [92,93,94]. HEK 293 cells were initially derived in 1973 from a kidney [95]. They are used as a functional tool to express recombinant proteins. HEK-293 cells are effective at transfection of nucleic acid and protein production [96]. Additionally, this cell line has a similar molecular pattern to cells of a neuronal lineage, and can thus be used in a neural context. HEK293 cells express 3 out of 4 major neurofilament subunits, other neuronal proteins, and neural enzymes. The cells also express endogenous voltage-activated ion currents similar to neurons [97]. Therefore, HEK293 cells can be used to mimic neurodegenerative diseases [93,98].

Therefore, the novel trio-drug combination of SVP (150 mg/kg) with BFN (5 mg/kg) and THQ (40 mg/kg) exhibits potential anti-epileptic, anti-inflammatory and neuroprotective effects, thus could be investigated in clinical studies as novel therapy for epilepsy.

## 5. Conclusions

Polytherapy is an important weapon in the arsenal against pharmacoresistant epilepsy. Studies have shown increased treatment persistence and reduced risk of hospitalization in a large population study of 8617 patients using combined AEDs of different mechanisms to control focal seizures. Furthermore, seizure control in any way possible is important, as uncontrolled episodes of epilepsy in those with drug resistant epilepsy can lead to a 2 to 10 times greater risk of sudden death compared to the general population. We report the anti-convulsant and neuroprotective potential of the trio-drug combination of SVP (150 mg/kg) + BFN (5 mg/kg) + THQ (40 mg/kg). The mechanism involved seems to be the reversal of the seizure-induced rise in the levels of inflammatory markers and mTOR. The results were supported by results from cell culture and in-silico studies. These results have important implications in the treatment of patients with generalized type of seizures and in those in which the higher doses of Sodium Valproate are not tolerated (as sodium valproate is hepatotoxic).

## Data Availability

Not applicable.
